# Multiplatform Mobile Laser Scanning: Usability and Performance

**DOI:** 10.3390/s120911712

**Published:** 2012-08-29

**Authors:** Antero Kukko, Harri Kaartinen, Juha Hyyppä, Yuwei Chen

**Affiliations:** 1 Department of Remote Sensing and Photogrammetry, Finnish Geodetic Institute, P.O. Box 15, FI-02431 Masala, Finland; E-Mails: Harri.Kaartinen@fgi.fi (H.K.); Juha.Hyyppä@fgi.fi (J.H.); 2 Department of Navigation and Positioning, Finnish Geodetic Institute, P.O. Box 15, FI-02431 Masala, Finland; E-Mail: Yuwei.Chen@fgi.fi

**Keywords:** multiplatform, mobile, laser scanning, 3D, mapping, accuracy, performance, test field

## Abstract

Mobile laser scanning is an emerging technology capable of capturing three-dimensional data from surrounding objects. With state-of-the-art sensors, the achieved point clouds capture object details with good accuracy and precision. Many of the applications involve civil engineering in urban areas, as well as traffic and other urban planning, all of which serve to make 3D city modeling probably the fastest growing market segment in this field. This article outlines multiplatform mobile laser scanning solutions such as vehicle- and trolley-operated urban area data acquisition, and boat-mounted equipment for fluvial environments. Moreover, we introduce a novel backpack version of mobile laser scanning equipment for surveying applications in the field of natural sciences where the requirements include precision and mobility in variable terrain conditions. In addition to presenting a technical description of the systems, we discuss the performance of the solutions in the light of various applications in the fields of urban mapping and modeling, fluvial geomorphology, snow-cover characterization, precision agriculture, and in monitoring the effects of climate change on permafrost landforms. The data performance of the mobile laser scanning approach is described by the results of an evaluation of the ROAMER on a permanent MLS test field. Furthermore, an *in situ* accuracy assessment using a field of spherical 3D targets for the newly-introduced Akhka backpack system is conducted and reported on.

## 1. Introduction

**Figure 1.**
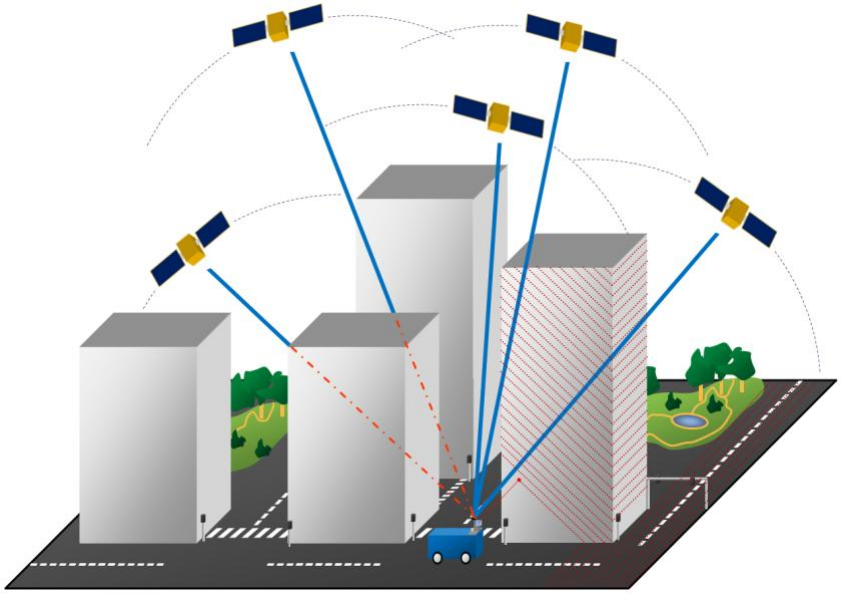
Mobile laser scanning utilizes GNSS-IMU positioning for direct geo-referencing of laser scanning data for three-dimensional mapping of objects.

Increasing interest has been shown in vehicle-based (mobile) surveying applications of laser scanning since the beginning of the 21st century when laser scanners began to be incorporated in what may be called mobile mapping systems (MMS) [1]. Mobile laser scanning (MLS) is a rapid and flexible method for acquiring high-resolution three-dimensional topographic data. MLS systems are lidar-based mobile mapping systems, which produce three-dimensional point clouds from the surrounding objects using profiling scanners; however, new types of scanners are emerging into the market. The spatial coverage is achieved by the movement of the vehicle and motion-tracking navigation devices, as illustrated in Figure 1. The survey is conducted as the ground vehicle moves around while the navigation system, typically based on a global navigation satellite system (GNSS) and inertial measurement unit (IMU), tracks the vehicle's trajectory and attitude for producing a 3D point cloud from the range data collected by the onboard scanners. Analogous to airborne laser scanning (ALS), the characteristics of the obtained point cloud, e.g., density, point pattern, and distribution, depend largely on the sensor arrangement on the platform, and the sensor properties, such as point measurement rate, scan frequency, and wavelength (e.g., [2–4]). Different layouts and approaches have been reported in numerous papers, e.g., [3,5–10].

Mobile mapping is expected to provide ease of mobilization and low costs when compared to airborne laser scanning. These points are especially attractive for projects involving small areas and specific tasks. In addition, the sensor layout of an MMS and other surveying arrangement can be adjusted more freely in comparison to the ALS to meet task-specific requirements. Furthermore, stationary data collection has some weaknesses: poor efficiency in data acquisition, difficulty of planning for viewpoints and directions in data acquisition when measuring large and complicated scenes, and the complexity of a registration method capable of succeeding in automated registering of all kinds of range data [11,12].

The applications of MLS to environmental remote sensing have thus far focused on vegetation and erosion studies and hydrology [13–16], while a number of applications has been presented for urban road environments [1,9,17,18]. The MLS systems are capable of faster and more efficient 3D data acquisition than stationary terrestrial laser scanning (TLS), especially in cases where ground validation (e.g., small-scale details) is needed for purposes such as airborne experiments or when dealing with areas covered by satellites observing the Earth (see [19,20]).

Most of the mapping applications in various fields stand to benefit from the accuracy and efficiency of MLS technology. Compared to traditional mapping methods, which utilize digital aerial images and airborne laser scanning, the precision of the data collected can be greatly improved. Furthermore, the time and cost of geodetic measurements with total stations and terrestrial lasers can be reduced. Beyond that, numerous advantages arise when using MLS data to produce high-resolution 3D models. This is demonstrated by the application examples in Section 2. Considering data acquisition compared to data from stationary terrestrial laser scanning, MLS provides high efficiency and a precise way for generating dense point clouds, and mobility makes it more suitable for surveying and modeling of large areas. 3D models processed from the data collected by MLS offer high-resolution visualization and surface analysis, which cannot be achieved from ALS and/or aerial images since they provide coarser rendition with considerably lower point density and precision.

The current version of the ROAMER, a single-scanner mobile mapping system for road environment mapping, was launched internationally in 2007 [8]. It enables the operator to use vertical or tilted scanning planes for adapting the system for appropriate 3D point acquisition in the carrying out of different tasks. The latest scanner version of the system operates with a point measurement frequency of up to 976 kHz and a maximum profile measurement rate of 61 Hz, and an ambiguity interval in the phase-shift ranging of 153 m. The relative point precision of the system is estimated to be below 1 centimeter, but its absolute accuracy is mainly dependent on the GNSS-IMU navigation solution that can be provided in real-time, or more reliably through post-processing by means of a tactical-grade GPS-IMU with an output of 100 Hz. In the work described in this paper, we used the multiplatform approach to operate the ROAMER, and we describe and discuss the completely new Akhka backpack MLS system. The paper also presents results from an evaluation of the data accuracy of the ROAMER on a permanent MLS test field, as well as results from an accuracy assessment of the Akhka data against implemented *in situ* target field method and TLS reference data.

## 2. Multiplatform MLS

**Figure 2.**
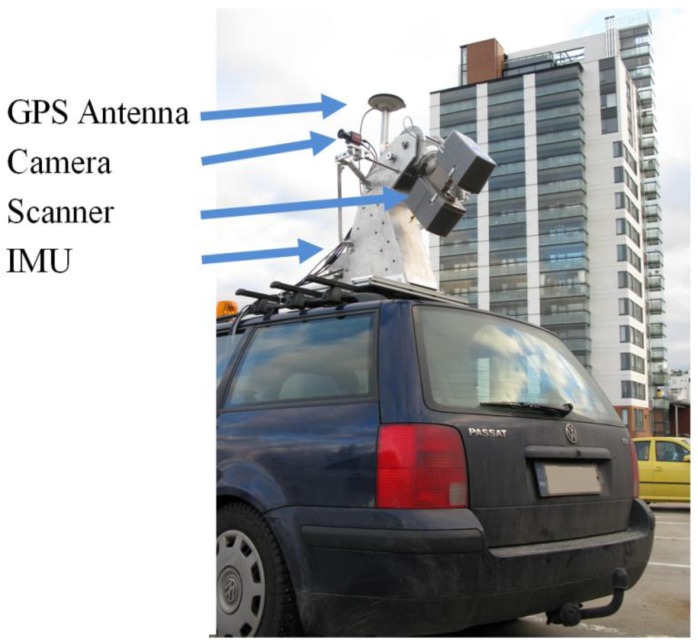
ROAMER as a vehicle-mounted MLS installation (Photo courtesy of H. Hyyppä).

The FGI's ROAMER mobile laser scanning system, Figure 2, is a high-end surveying device for producing accurate, dense, and precise point clouds for three-dimensional mapping for the detection, localization, modeling, analysis, and monitoring of anthropogenic and natural phenomena and processes. The initial goal of having this system was to develop a system that would maximize the automation of feature extraction at the post processing phase [8]. To accomplish a high level of automation in data processing, a laser scanner capable of providing dense point clouds was set as the requirement for the system. Additionally, the system was required to be a moving laboratory flexible as regards various applications. By upgrading the scanner unit several times, the data needs of different surveys have been met. The system performance has been improved by continuing with the development of processes and updates of hardware during the course of the past five years.

Table 1 summarizes the current ROAMER equipment and the main data acquisition parameters that are operator-selectable to enable adapting the data acquisition to different tasks. The ROAMER system is a DC-powered compact unit that can be installed on various carrier platforms for mobility and to meet the requirements set by various applications. The system runs on battery power for several hours at a time, whereas when mounted on vehicles the vehicle's DC system can be used as its power source for continuous operation. The data recording computers are rugged laptops; one for navigation and the other for the laser scanning data.

The laser scanning unit in ROAMER is a FARO Photon 120 that uses a 785 nm laser with a power of 20 mW (Laser class 3R). The laser beam diameter at the beam exit point of the scanner is 3.3 mm and the beam spreads according to 0.16 mrad divergence angle. This results in a laser footprint with a size of 20 mm at 100 m from the scanner; this, together with precise range measurement, enables detailed 3D measurements to be made of objects. The highest available angular resolution with a scanning frequency of 48 Hz is 0.3 mrad (0.018°); with PRF of 976 kHz and with 61 Hz scanning frequency, the corresponding value is 0.8 mrad (0.045°) with PRF of 488 kHz. Ultimately, the point distribution on the object surface depends on platform velocity, surface orientation, scanning angle, and object distance from the scanner.

In a typical MLS project with the ROAMER, Waypoint Inertial Explorer™ GPS-IMU post-processing software is used for computing the system trajectory. The software combines the reference station data to the GPS and IMU data collected by the SPAN equipment on-board the moving mapping unit. The computed trajectory solution is combined with the raw laser data with system boresight calibration information to compute the 3D point clouds.

### 2.1. Vehicle MLS for Urban Mapping

Vehicle-mounted MLS is currently widely used in connection with mapping of urban areas, as the vehicle platform provides sufficient speed in pace with the traffic, and enough room and elevation for the surveying equipment. Large datasets of urban objects can be produced using MLS techniques and various surveying tasks can be performed using the dense and precise MLS data point cloud.

**Figure 3.**
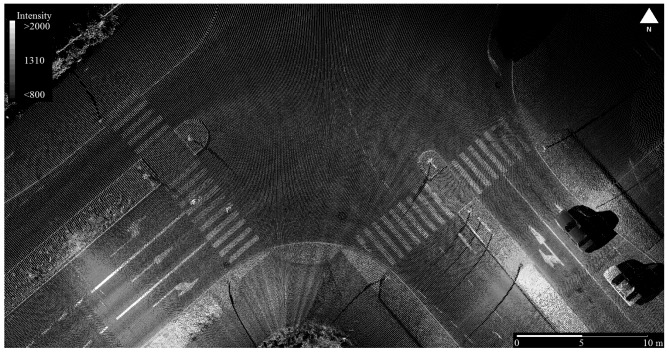
Vehicle-mounted MLS data with intensity-scale coloring of a street corner; besides street geometry, intensity data enable identification of road paintings such as zebra crossings.

Vehicle-mounted MLS street data typically contain intensity data, which can be used to automatically extract road paintings, e.g., zebra crossings, and geometric information about buildings, pavement, pedestrian structures, and islands, manholes, curbs, poles, signs and pylons, as shown in Figure 3. Intensity readings can be utilized in conjunction with geometric data for automatic extraction of different target types from the point clouds [17]. Detection and inventory of utility poles, traffic signs and lamp posts are prime examples of utilizing MLS in urban infrastructure maintenance, e.g., [18]. MLS data can also provide up-to-date information on power lines and other open-air infrastructure, railway facilities, *etc.*

The scanning geometry and point density for a mobile lidar are different from those for airborne laser scanning. The point spacing of adjacent points in a profile projected on a plane perpendicular to the beam at the typical range of 20 m in urban mapping is thus 6 mm for a scanning frequency of 48 Hz and 16 mm for a scanning frequency of 61 Hz. This is sufficient for 3D modeling purposes at any resolution level within the scan profile. Along-track profile spacing is less than 0.5 m even for speeds up to 70–80 km/h when high mirror speeds are used. For platform speeds of 50–60 km/h, a profile spacing of around 1.0 m is achieved also for the mid-frequencies of mirror revolution. With a scanning frequency of 48 Hz, the profile interval becomes better than 25 cm when the speed of the mapping unit is kept under 40 km/h, and even then the point resolution along the profile is 2–5 cm, which is sufficient for practical ranges of 20–40 m, respectively, in the urban environment. One factor influencing the distribution of the laser points on the scene is whether the scanner is vertical or has been placed in one of the tilted positions. Further performance upgrading of the ROAMER system can only be achieved by increasing the scan frequency.

Tilted scanner positions are mostly used to obtain points from the road surface while bearing in mind the scanners 320° field of view. The tilted scanning plane also produces multiple hits even of narrow pole structures usually present on the both sides of the road, e.g., traffic signs, light poles, and bridge pylons. This is a function of scan frequency and point density along the profile, and due to the fact that the wide FOV of the scanner makes it possible to acquire multiple hits from several sequential profiles from an object as the mapping unit passes by. Such an approach enables higher platform speeds than when using vertical profile orientation that may completely miss narrow vertical structures. As the only non-fixed variable during the survey is the speed of the vehicle, it ultimately determines the number of profiles sweeping the object. The positioning and modeling of the object becomes more reliable with multiple point arcs; this is demonstrated by [18].

A further advantage of using the tilted scanning plane is the ability then of capturing vertical and horizontal edges with equal, angular-resolution-dependent sampling, independently on profile spacing; see [12] for more details. Such objects include corners of buildings and driveways, and window and door frames in the facades of building. Moreover, at a corner of a building, the laser beam illuminates portions of the two facades of the building, and thus data points are measured from the two walls. This helps in more reliably locating the building corners from the point data. This also applies to window frames for more detailed surveys. With equal scan frequency, angular resolution and velocity, tilted scanning provides more information about the object along the track direction than vertical scanning. Thus, platform velocity and scan frequency have greater impacts on the data pattern, *i.e.*, point distribution, when using systems with a vertical scanning plane.

### 2.2. Trolley MLS for Restricted Areas

**Figure 4.**
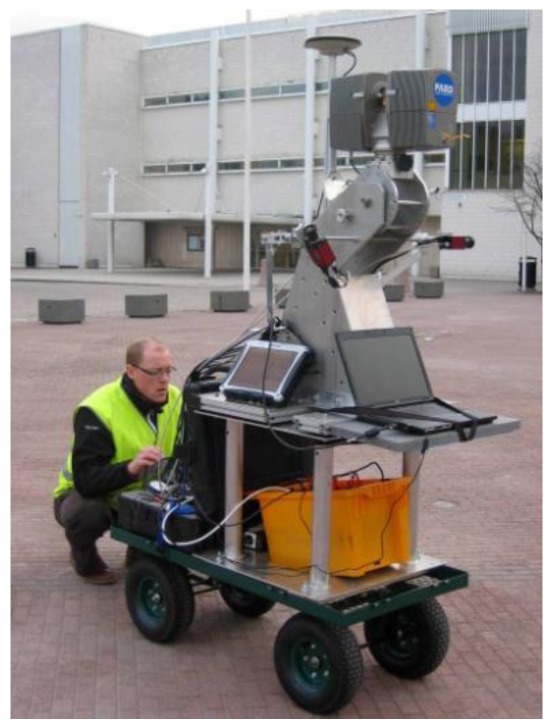
Initializing the cart carried MLS in urban mapping.

Some environments and application tasks are such that a motorized vehicle is not practical due to its weight, dimensions, noise, and exhaust gases. For such situations, the ROAMER MLS can be mounted on a trolley. A trolley provides an easy-to-handle and steady transportation of the MLS unit in and around the area to be mapped, e.g., urban pedestrian areas and farmland, as is shown by Figure 4.

Urban modeling is the field into which global enterprises focusing on 3D products, such as Nokia NAVTEQ, Microsoft and Google, are investing a lot of efforts. Mobile mapping is the only technology to offer the pedestrian point of view with sufficient level of detail for personal navigation using mobile handheld devices such as those introduced in the 3D-NAVI-EXPO project [21].

**Figure 5.**
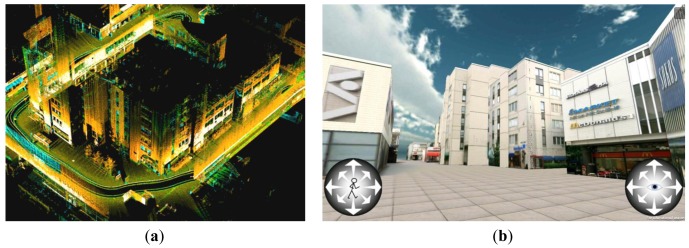
(**a**) A three-dimensional point cloud; (**b**) a photorealistic model of Tapiola, Finland, processed from MLS data and digital imagery (Image on the right by courtesy of A. Jaakkola).

Tapiola is one of the main suburban centers of the City of Espoo, in southern Finland, and it was selected as a test area for mobile navigation research conducted by the FGI. Mobile laser scanning data were acquired on May 12th, 2010 using a ROAMER on a trolley with vertical scanning, which provides good coverage of building facades along narrow passages and when making steep turns. The scanner head was set to capture 244,000 pts/s with 48 Hz mirror scan frequency. The GPS reference station data were acquired from the Finnish virtual reference station (VRS) network. The Tapiola MMS data include a total of some 160,000 profiles; with each profile having 5,000 points with a 3D-position and a return-intensity-producing point cloud; a section of this is seen in Figure 5(a). The data collection lasted about one hour and it covered an area 200 m × 300 m in size.

The models were reconstructed mainly in two steps: (i) Fully automated geometry reconstruction from raw laser data to produce buildings and to enable corner detection. This step included also interactive model checking and refining using software for building geometry; (ii) Photo realistic texture preparation and mapping. To produce the final model, the image data were taken separately for the textures of building facades because of high buildings and narrow streets. The images taken by the ROAMER system did not cover all of the building facades in the case of high buildings and these images did not meet the photographic requirements for high-quality textures. The delineation of the automatic processes used in the model point data manipulation is described in detail by [22]. The navigable Tapiola model is available on the Android Market as a free download (http://market.android.com/details?id=com.FGI.Tapiola3D).

### 2.3. Fluvial Geomorphology Research with MLS Installed on a Boat

**Figure 6.**
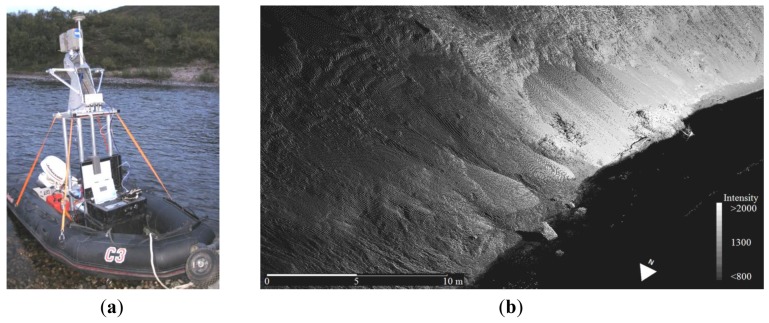
(**a**) A boat-mounted MLS for mapping of fluvial processes; (**b**) 20 m high river bank subject to flood erosion mapped using a boat-mounted MLS. Geomorphological features are easily detected due to the dense point clouds.

The first non-urban application of the ROAMER was to install the system on a boat as shown in Figure 6(a). Having a fast laser scanner, it enables detailed riverine topographical data to be acquired for fluvial applications such as hydraulic modeling and geomorphology. In addition to static modeling of riverine topography, there is growing need to map changes in topography as the geomorphology and topography of the river channel and surrounding floodplain are affected by fluvial erosion and deposition processes, which vary from constant grain-scale displacement to large-scale flood-related avulsions [23–25].

In the boat installation, a.k.a. Boat Mobile Mapping System (BOMMS), the laser scanner was elevated approx. 2.5 m above the water surface by means of a stand and vertical scanning was employed to yield adequate measurement geometry when considering the flat point bar areas. Combined with terrestrial laser scanning data (in the 2008 campaign), boat-mounted mobile laser scanning facilitated a whole new field mapping approach for fluvial studies [14]. The mobile mapping approach proved to be an extremely rapid method for surveying riverine topography, taking only 85 min to survey a reach approximately 6 km in length with typical profile density of 40 m^−1^. The approach also enables an effective survey angle for deep river banks. This is difficult to achieve when using airborne or terrestrial scanning. The BOMMS system has been used in data acquisition in numerous studies involving hydraulic modeling and analysis of geomorphological processes during the years 2008–2011 (e.g., [14–16]).

The results obtained in fluvial studies using the BOMMS indicate that the MLS could provide accurate and precise change information over large areas. However, the data need to be controlled for systematic errors, as these significantly affect the volumes derived from surface analysis. Sufficient reference can, for example, be carried out by setting specific targets in the surveying area and by providing additional ground reference points using conventional surveying techniques (RTK-GPS, TotalStation).

### 2.4. Precision Farming: Leaching Field DEM

A light weight MLS (around 50 kg) was applied in a study case to generate a high-resolution digital elevation model (DEM) of a leaching field belonging to Agrifood Research Finland and situated in Toholampi, Western Finland. The field consists of 16 plots of 1,600 m^2^ each. The soil type is fine sand, with 5% organic carbon in the ploughing layer. The field has been part of an experimental setup with organic and conventional crop rotations since 1997. The plots are sub-drained and water is collected separately from each plot and led into the monitoring building, where the volume of water is measured and flow-weighted water samples for analyses are taken manually. Surface water is also collected and measured.

The aim of the case study was to collect dense MLS (mobile laser scanning) data for producing a high-resolution ground surface model of the leaching field. The ROAMER mobile laser scanning unit was employed for the task. The surface data were collected in May 2011 with the MLS on a trolley to avoid damaging the new crop. The scanner head was elevated 1 m higher than in the Tapiola data acquisition exercise, and it was angled to point 45 degrees below the horizon to capture the ground. Otherwise the scanning parameters were the same. The survey took about an hour with the GPS-IMU initializations.

**Figure 7.**
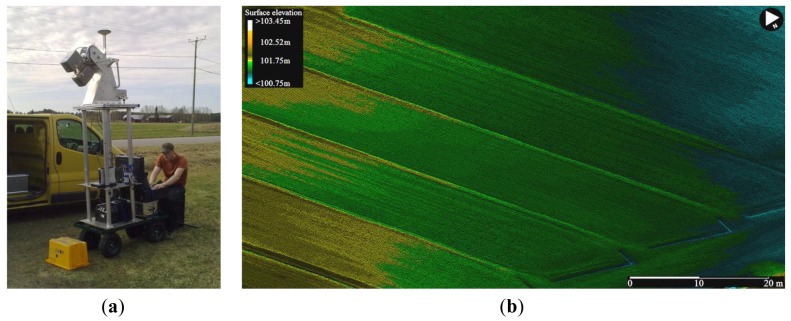
(**a**) An MLS setup for the leaching field experiment; (**b**) Ground surface topography could be reproduced from the MLS point cloud with a high level of detail (10 cm DEM).

Figure 7(b) shows an extract of the 10 cm DEM data processed from the point clouds. Separate field patches can be seen, including the seed drill traces and some of the drains for collecting the surface water. High point density thus enables detailed analysis of fine-scale ground elevations. This case shows, in addition to what was reported in the fluvial case above, that accurate high-resolution ground surface models can be rapidly produced with the MLS.

### 2.5. All-Terrain MLS in Forestry and Snow

**Figure 8.**
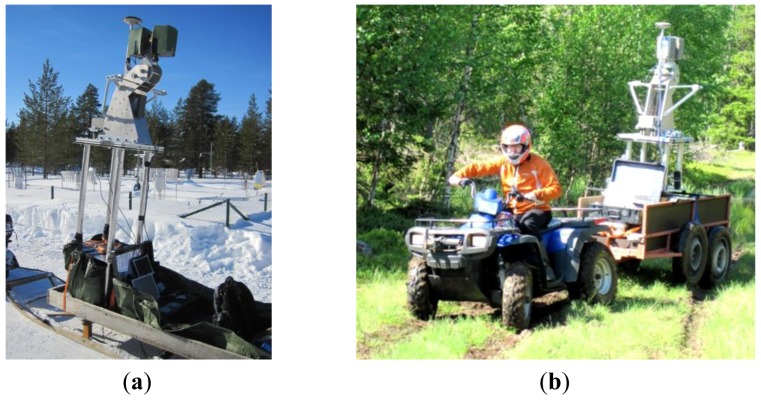
(**a**) A snowmobile application for studying the characteristics of and changes in snow cover; (**b**) an all-terrain vehicle towing the MLS when mapping in non-snow conditions.

The ROAMER system was utilized for three-dimensional snow data capture in the 2010 and 2011 campaigns. The campaigns were conducted for snow surface characterization required for airborne and satellite-based snow measurement validation. In this application dense point clouds were acquired from a snow mobile installation. The weather conditions on the site where the system was operated were dry and a few degrees below zero. The scanner unit was covered with thermal insulation to help retain the operational temperature of the laser head. Despite the unconventional operating conditions, the data acquired show no degradation in quality or coverage. Figure 8 shows the arrangements for snow surface and ground free of snow in connection with the MLS data collection in the spring thaw and summer seasons in 2011.

**Figure 9.**
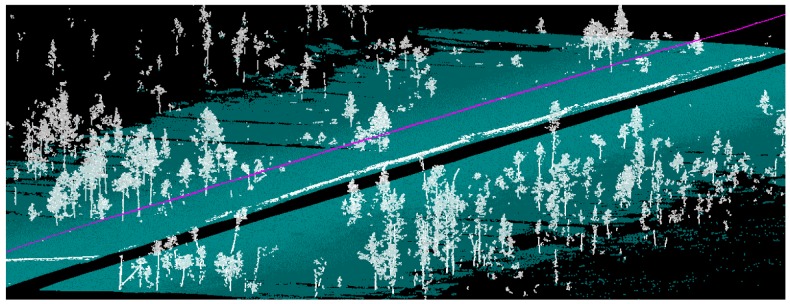
Snow surface and trees detected by classifying the MLS data. The purple line shows the scanner trajectory.

This arrangement facilitated the production of dense, precise, multi-temporal point cloud data over wide areas covered by snow (Figure 9). The data acquired in the course of each campaigns consisted of several strips of point data from study sites around Sodankylä Arctic Research Center. The longest stretch of continuous data was about 11 km long with the multi-temporal data acquisitions conducted usually on subsequent days. By utilizing the MLS, the spatial coverage and statistical variation in the data was dramatically improved when compared to traditional methods. The ease of application of the proposed system enables repeated surveys even for short-term analysis on a daily or hourly basis.

The multi-temporal snow surface data enables one to study the changes in snow depth [26] and snow surface topography and roughness [27]. The MLS approach is capable of providing multi-scale data from millimeter to several meters in the vertical direction, and from the centimeter scale, mainly restricted by profile spacing, to tens of meters (and even kilometers) in the horizontal direction. Based on the experience from the two seasons in 2010–2011, applying the MLS in seasonal snow research appears to be a practical solution and opens up new possibilities for snow surface characterization. Mobile laser scanning provides better possibilities for statistical analysis of the snow surface roughness and its impact on the surface albedo than do traditional field methods, the latter being labor intensive and thus often spatially limited. In our on-going research, we use MLS data for computing fine-scale surface roughness in connection with different ground sampling densities and sizes, ranging from centimeters to hundreds of meters, and then evaluate the results [27].

### 2.6. Akhka-Backpack MLS for Mobility

**Figure 10.**
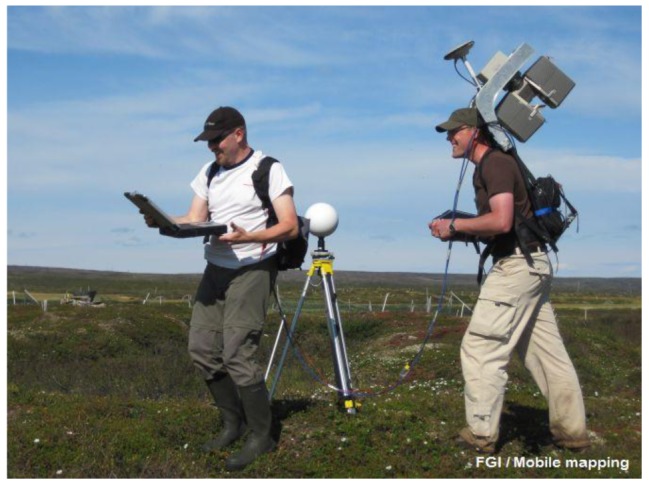
Mapping of permafrost palsa landforms in Finnish Lapland with the Akhka backpack mobile laser scanning unit.

A whole new approach for MLS was needed in a study case involving the monitoring of arctic permafrost palsa landforms from the viewpoint of climate change. One such study was conducted on the Vaisjeaggi palsa mire close to the Kevo Research Station, Utsjoki, in northernmost Finland. An innovative backpack solution was constructed to meet the challenges of mobility on the mire and it was named *Akhka*. The backpack platform provides mobility and high-performance 3D surveying capacity in environments where wheeled vehicles cannot be used due to the weak carrying capacity of the substrate, narrow passages or other such limitations. The scanner head in the Akhka solution is mounted directly under the IMU unit and it points downwards roughly at an angle of 40 degrees to yield cross-track profiles; see Figure 10.

We used the Akhka backpack MLS system to map a palsa landform area measuring 50 m × 100 m in June and September 2011 to test the system's operability and to analyze its capability in producing high-resolution multi-temporal DEMs for change analysis for a climate change network. In the first field test, the Akhka MLS was operated by a crew of two, but work is going on to miniaturize the system to make it operable by one person. Currently, most of its 25 kg weight comes from the batteries and the scanner unit, which could be replaced with lighter ones in the future. However, the surveying capacity of the Akhka is essentially equal to that of the ROAMER as the sensors are the same (see Table 1) and only the integration platform differs.

The offsets between the scanner, the IMU, and the antenna phase center were measured in laboratory conditions with an estimated accuracy of 2 mm. The boresight angles between the IMU and the scanner were determined by estimating them from the systematic discrepancies in the sphere target observations in relation to the scanner trajectory.

**Figure 11.**
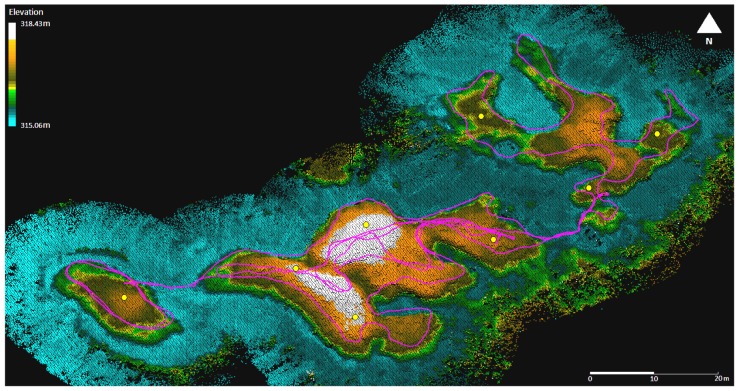
An elevation model with a 20 cm grid size of Vaisjeaggi permafrost palsa landform surveyed with an Akhka backpack MLS in June 2011. The survey trajectory is represented by purple lines and the control spheres by red circles.

Figure 11 shows the point cloud obtained in June 2011 with coloring for elevation inserted after processing. The scanner trajectory is illustrated at top of the point data as a purple line. The point density over the area of interest varied from 1,800 pts/m^2^ to 50,000 pts/m^2^, with the mean point density being 9,100 pts/m^2^.

The geometric quality of the point cloud data was verified against eight spherical targets erected on top of the palsas and located by means of RTK-GPS. Three scans with a Leica HDS6100 were acquired for validation of the data and these were geo-referenced using the spherical targets. The mean mismatch between the RTK-GPS and TLS data was 15 mm with a standard deviation of 7 mm. The maximum deviation between RTK-GPS and TLS scan registration was 32 mm at Target #6. In Section 3.2 we show that this target also exhibited discrepancies in the other tests. The geometric analysis is reported in more detail in Section 3.2 of this article.

To the best of our knowledge, the Akhka is a world-first and a very promising approach to high-performance MLS, although more generally backpack platforms have been deployed before [7,28]. The backpack version expands the range of applicability of mobile laser scanning technology by opening up new possibilities into research fields that have lacked detailed 3D surveying capability and areal coverage. The presented study case is a good example of that. We see that such MLS systems possess good potential in speeding up and intensifying the collection of 3D survey data and thereby widening spatial coverage with remarkable point density and data quality. Future applications could make their contributions in various modeling tasks in the vast fields of civil engineering, archaeology, and geoinformatics, as well as in monitoring and understanding of processes in different disciplines of natural sciences such as cryosphere (e.g., the monitoring of seasonal snow coverage) and glaciology, geography, hydrology, silviculture, and agriculture.

## 3. Performance Analysis of the ROAMER and the Akhka MLSs

Experiences gained from earlier research have shown that permanent test fields with accurate ground truth are valuable tools for analyzing the performance of remote sensing systems and methods in mapping tasks. The performance of the ROAMER equipment was verified on a MLS test field using an EuroSDR comparison [29]. The mobile mapping test site set up by the Finnish Geodetic Institute is located about 16 km west of Helsinki, Finland. The test site consists of one block covering 1,700 m of urban road environment with different segments having varying GNSS visibility.

The Akhka system utilizes the same navigation and data capturing equipment as the ROAMER (see Table 1). Thus, the performance of the systems can be expected to correspond to each other. However, the ground velocities for Akhka-based data collection are typically much lower than those for the vehicle-mounted ROAMER, and abrupt turns occur more frequently. A rugged terrain also adds abrupt vertical accelerations to the IMU data. All these factors may have impact on the quality of the navigation solution, and thus an *in situ* field calibration and control scheme was implemented for data quality assessment in the field.

### 3.1. ROAMER vs. FGI Permanent MLS Test Field

**Figure 12.**
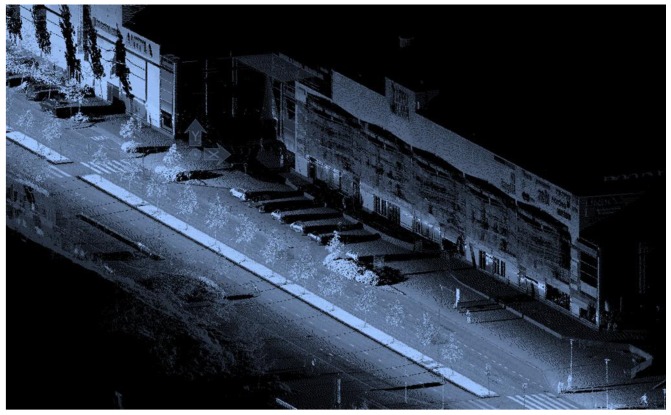
The ROAMER test data obtained on the FGI MLS performance test field.

To enable accuracy assessment, the ROAMER was placed on top of a vehicle and the test site was surveyed by driving the test route in two directions (Clockwise, CW, and counter-clockwise, CCW) in June 2009. At that time, the ROAMER consisted of a Faro Photon 80 terrestrial laser scanner and NovAtel SPAN positioning system (NovAtel DL-4 plus GPS-receiver, NovAtel GPS-702-GG antenna, and Honeywell HG1700 AG58 IMU with ring laser gyros). The maximum point measurement rate of the Photon 80 scanner was 120 kHz and the maximum range ambiguity was 76 m. Laser profiling was carried out using a scan frequency of 48 Hz, and the scanner was arranged to measure profiles tilted to 45 degrees below horizontal to produce the point cloud seen in Figure 12.

Firstly, the ground points were classified and a regular grid with 5 cm point spacing was computed to achieve an even distribution of the ground points. This grid was thinned by selecting every 1,000th point, and these thinned points were compared to the original ground points. All thinned points deviating more than 5 mm from the original data were deleted, and the remaining points were selected as reference points for analysis of elevation accuracy. The complete ground reference data for the elevation analysis consisted of 3,283 points; also the distance and direction to all possible driving trajectories were determined.

A set of targets was measured from the reference data for analysis of planimetric accuracy from an area 350 m long within the test site and having virtually the best GPS visibility. The targets included centers of poles, building corners, and curb corners. The pole center coordinates were measured by visually fitting a circle to the point cloud as seen from above. Altogether 273 planimetric reference targets were measured to analyze the system's accuracy.

#### 3.1.1. Detecting Gross Errors and Compensating for Systematic Shifts

The elevation points showing the highest discrepancy were checked against the ground truth and deleted from the analysis if the error was due to the target, but not due to the system. These errors were mainly found to occur due to changes in the environment between the test field data collection and MLS data acquisitions (e.g., cars, vegetation, and the shadows caused by these). Next, the systematic errors were computed as averages of residuals along the three coordinate axes and the MLS data were compensated for as summarized in Table 2. The proper treatment of systematic discrepancies is essential for accuracy assessment; this is the case especially as large systematic shifts in a plane can lead to distorted elevation accuracy results. The remaining errors were assumed to be system-specific, *i.e.*, calibration, positioning and scanner related.

#### 3.1.2. Assessing System-Specific Errors

Subsequent to systematic error compensation, system-specific error values were computed. Minimum, maximum, and standard deviation values were computed for both elevation and planimetric estimation. Mean and RMSE (Root Mean Squared Error) were also derived for planimetric accuracy.

A comparison between the elevation reference points and the ROAMER point clouds was carried out using the output control report tool in TerraScan software [30]. It reads in the reference points and loads every laser point within a given search radius from the individual reference point. Then a small triangulated surface model is created from the laser points and elevation is estimated for each reference point from the triangulated model surface. This effectively interpolates the required laser elevation from three laser points, which are closest to a given reference point. The search radius used in the computation was 50 cm. The maximum permitted slope in the triangulated model was set to 20 degrees, as steep slopes are generally not ideal when estimating the elevation error.

The results of the elevation accuracy analysis are summarized in Table 3; there separate error figures are given for each of the opposite driving directions (CCW, CW). The elevation accuracy was analyzed at approx. 2,800 check points resulting in a standard deviation of 20 mm.

The planimetric accuracy was evaluated by measuring the reference targets within the MLS point clouds and computing the differences in easting and northing. The reference targets observed were typically building corners, lamp post and other pole center locations, and curb corners. The results of the planimetric accuracy analysis are presented combined in Table 3. The standard deviation shows pretty good figure for the ROAMER system's precision, and the RMSE level is a pleasing indicator of absolute accuracy.

**Figure 13.**
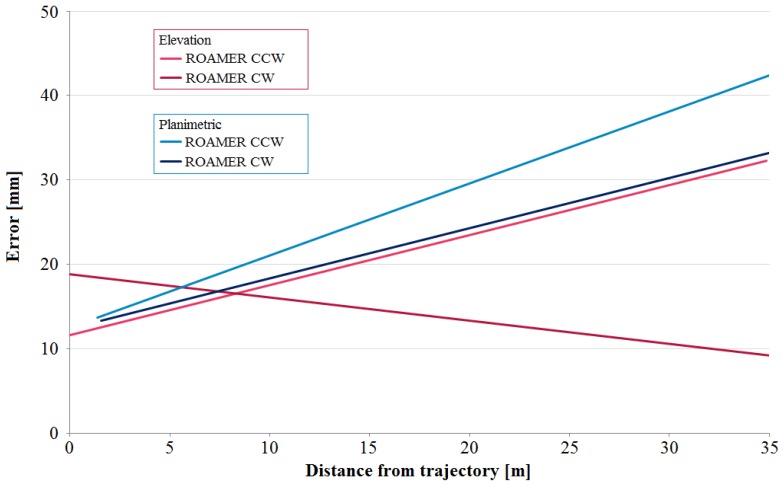
The elevation and planimetric errors as functions of distance from the trajectory for the two driving directions.

Elevation and planimetric accuracies expressed as functions of target distance from the trajectory are shown in Figure 13. The linear trend lines fitted to observed errors suggest that the system's boresight calibration, *i.e.*, the alignment of the IMU with respect to the laser sensor, still has some minor erroneous systematic behavior. The slope of the trend lines describing the planimetric error show that the error in heading angle is approx. 0.033° (0.6 mrad). The elevation trend enabled us to estimate a systematic error of 0.023° (0.4 mrad) in the roll angle. These results suggest that quality calibration requires calibration observations also from the far field of the scanner (long-range observations) with adequate localization.

#### 3.1.3. Factors Affecting MLS Accuracy

As the results show, given good GNSS coverage, the ROAMER system is able to acquire accurate point cloud data with extremely good accuracy and resolution. It often happens that buildings, trees, and other structures obstruct satellite visibility and the performance of other navigation instruments, such as IMUs and odometers, as well as post-processing algorithms define the achievable accuracy. Tools for improving trajectory accuracy are being developed and new satellites are being launched, both of which should improve accuracy in areas where the current GNSS-IMU based mobile mapping systems run into problems.

The result further indicates that system calibration has a major impact on performance. By implementing a field calibration scheme, *i.e.*, using test field TLS data in estimating the bore-sight parameters of the ROAMER, accuracy can be improved considerably. When compared to the first point cloud data computed without improvements from the field calibration, the standard deviations of both elevation and planimetric errors were diminished by half; see [28] for further details. Errors in the relative orientation of the instruments lead directly to errors in the measured point clouds, which cause problems in continued manipulation of the data, e.g., in extraction and modeling of objects.

### 3.2. Akhka vs. Field Reference

Eight spherical reference targets (ATS Scan Reference System), 198.8 mm in diameter, were erected around the study area in connection with the September 2011 data acquisition for the purpose of assessing the quality estimates for the Akhka data in the field. The location of each sphere center was measured with RTK-GPS with an approximate base line length of 600 m from the bench mark with GPS base station. According to one report, RTK-GPS can, by default, provide 10 mm + 1–2 ppm horizontal and 15–20 mm + 2 ppm elevation accuracy for the target locations [31]. The spheres were also scanned with a Leica HDS6100 terrestrial scanner with a resolution of 0.036° (0.6 mrad, HIGH setting) from three locations to provide internal dimensions for the target field, and also to produce reference data of the ground surface. The scans were located in a least squares estimation according to the sphere coordinates from the RTK-GPS positioning.

**Figure 14.**
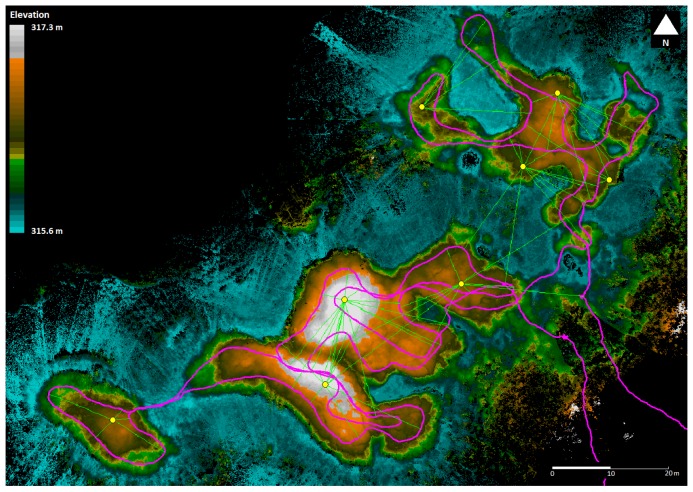
Assessing MLS data accuracy with spherical targets (red circles).

The internal precision of the Akhka system was analyzed against the sphere locations extracted automatically from the data each time that the spheres were detected in the field of view of the scanner. A model sphere 198.8 mm in diameter was matched by least squares estimation to the point sets to solve the target center location each time the MLS passed it. Figure 14 shows the target arrangement and extracted targets on top of the point cloud data. The purple line shows the MLS trajectory, and the green lines connect the targets pointed by the red circles to the trajectory locations from where they were detected. The point cloud coloring is inherent to the point elevation. The systematic errors found by comparing the coordinates of the sphere locations to the RTK-GPS data are shown at the bottom of Table 4. The 2D RMSE (Root Mean Square Error) for all the targets was 18 mm in the horizontal plane, and 29 mm in elevation so that the 3D RMSE for the targets was 34 mm. The values correspond closely to the result for the ROAMER case, showing even slightly better performance.

In Table 4, in summing up the error figures for each target, the “Mean” column describes the average absolute error of the MLS point cloud data against RTK-GPS observations, while “Standard deviation” shows the internal precision of the MLS data at each target location. “Maximum error” shows the largest deviation in the data for each of the targets. It is worth bearing in mind that the target distance from the scanner affects the accuracy. In this study, the target-to-scanner distance varied from 1.42 m to 19.95 m, being 9.72 m on average. In general, the error figures show good agreement internally as well as absolutely to the expected RTK-GPS accuracy levels of the target locations. The result reflects also the error estimation reported for the test field case with the ROAMER data.

We could see from the maximum errors that the largest planimetric error for Target #6 was 45 mm with 43 mm 3D target RMSE. The largest elevation discrepancy detected was 89 mm for Target #5, although Target #6 also showed exceptionally large elevation discrepancy when compared to the average. On the other hand, Target #8 appeared to have the largest mean elevation difference, which was an indication of problems in the navigation solution as all the observations for the particular target were only from over a short, 40 s, period of time. The internal precision of the target locations derived from the MLS data, however, indicated good performance; it was 11 mm in easting, 13 mm in northing, and 29 mm in elevation. The corresponding maximum deviations were 14 mm, 18 mm, and 40 mm.

The initial analysis of the ground elevation data derivation using the Akhka system was tested against the TLS reference scans. In order to be able to compare the appropriate data, 5 cm lowest hit point grids were computed from both the TLS and MLS data. The lowest hit points were further filtered for isolated points requiring points to be closer than 6 cm to any of the other points to reduce non-ground points from the analysis. The MLS data were also translated to correct for the systematic shifts found earlier for more adequate comparison.

After a subset of 2310 was filtered, TLS points were selected from locations close to the scan stations and where the surface roughness computed for the MLS data showed low roughness yielding thus higher probability of data expressing only bare ground observations (*i.e.*, avoiding low vegetation from the statistics). In the analysis, a systematic lowest hit ground elevation shift of 7 mm was found between the datasets, and the average magnitude of the mismatch was 14 mm. The standard deviation for the elevation in the data comparison was 16 mm, and RMSE was 17 mm. This shows that the data quality is, considering the non-obstructed GPS visibility, in good agreement with the results of the EuroSDR test for the ROAMER. However, a more thorough analysis of using MLS point clouds for digital elevation model generation on vegetated surfaces becomes emphasized. Also, a more thorough study on correcting the time-dependent variations in the trajectory based on the retrieved target data is something to be borne in mind for the future.

## 4. Conclusions

We have presented a variety of different platforms for mobile laser scanning applied to the 3D mapping of objects in different fields of human endeavor. The ROAMER MLS system has been employed in performing numerous of tasks… in urban environments, in agriculture, in projects dealing with geophysical and climate research. Although the measurement and navigation sensors used have been the same in all four approaches, the platform and settings have been altered to suit the different applications. This has been possible thanks to the relative lightness and compactness plus simple design of the system.

We have also presented the completely new Akhka backpack MLS platform for high-performance lidar mapping of areas where it is difficult, if not impossible, to operate with any ground-based vehicle. The Akhka system is built on a backpack pipe rig, and it enables exceedingly high point densities to be achieved with an absolute accuracy level of 20 mm. The Akhka is world-first and promising approach to high-performance MLS. It widens the range of applications of mobile laser scanning technology and enables new insights into research fields that have thus far lacked detailed 3D surveying systems with sufficient coverage, e.g., forestry and research focusing on fine-scale geographical processes.

It is evident that the proposed MLS approaches have the potential to speed up and improve the collection of 3D survey data and thereby widen spatial coverage with remarkable results as regards point density and quality. Future applications will have important parts to play in various modeling tasks in the vast fields of civil and transportation engineering, archaeology and geomatics, as well as in the monitoring and understanding of processes in different disciplines of natural sciences; e.g., cryosphere (an example of which is the monitoring of seasonal snow coverage) and glaciology, geography, hydrology, silviculture, and agriculture.

The performance of the proposed systems based on the analyses of results achieved on a permanent MLS test field and *in situ* target field studies show that the presented MLS systems can produce dense point cloud data for object reconstruction with absolute accuracy being at the level of some centimeters (horizontal RMSE 23 mm for the ROAMER and 17 mm for the Akhka) both in regard to plane and to elevation. The short-term relative precision of the data was estimated to be around 12 mm, provided that the internal calibration of the system is carried out appropriately. The error figures for the precision of elevation were found to be slightly less than double of the horizontal errors, being 14 mm and 29 mm for the ROAMER and Akhka, respectively. The elevation accuracy for the ROAMER data included a range-dependent systematic trend of 0.007 mrad. For multi- or hyper-temporal studies, the possibility of positional discrepancies should be taken in to account to ensure reliable analysis, and yet even at this level the advantages of incorporating MLS techniques represent a huge leap in change analysis applications in many fields. There is a need for more thorough analysis of using MLS point clouds for the generation of digital elevation model on vegetated surfaces. Also, a study on correcting the time-dependent variations in the trajectory, based on the retrieved target data, is a matter requiring addressing in the future.

Numerous authors have reported use of mobile scanners on different platforms, but no single group of authors has a yet reported an undertaking of this scope and variability. It has been shown through the application examples presented in this paper that when using an MLS system equipped with a high-performance laser scanner and sufficient navigation capacity, it is possible to produce accurate 3D point cloud data meeting application-specific needs in terms of data quality, density, and coverage. The benefits of using MLS data for producing high-resolution 3D models are obvious, as has been demonstrated in this paper. Considering data acquisition compared to the data acquired when using a stationary TLS, the MLS provides high efficiency and a precise way of generating dense point clouds, and its mobility makes it more suitable for surveying and modeling large areas. Intelligent design with an easy to adjust approach together with light data recording facilities are the keys to the versatility of the proposed equipment. Future research contributions will concentrate on the further development of automatic data correction and field control schemes, as well as on object modeling and surface analysis methods.

## Figures and Tables

**Table 1.** ROAMER MLS system equipment and characteristics.

FARO Photon 120 scanner 120,000–976,000 pts/s, selectable 320° maximum field of view 3–61 Hz scan frequency, selectable Wavelength 785 nm

NovAtel SPAN GPS-IMU NovAtel DL-4plus receiver and GPS-702 antenna L1 and L2 frequencies Honeywell HG1700 AG11 tactical-grade RLG IMU Gyro bias 1.0 deg/h Random walk 0.125 deg/rt-h Data rate 100 Hz

Data recording Panasonic CF-29 Scanner operations and recording Panasonic CF-19 Navigation system operation and recording

Bi-trigger synchronization Electronics built in-house Scanning start-stop Delivers scanner triggers to receiver log Camera triggering × 4

**Table 2.** Systematic errors for the ROAMER data. Driving directions: counter-clockwise CCW, and clockwise, CW.

	**Systematic error (mm)**	
	
	CCW	CW
Northing	14	25
Easting	5	0
Elevation	59	43

**Table 3.** Planimetric and elevation error values for the ROAMER MLS data. Driving directions: counter-clockwise, CCW, and clockwise, CW.

	**Planimetric error (mm)**		**Elevation error (mm)**	
	
	CCW	CW	CCW	CW
Number of reference	136	120	2819	2816
Mean	22	18		
Min	2	3	−68	−75
Max	67	51	60	50
Std	11	9	20	20
RMSE	25	20		

**Table 4.** The internal deviation of the target sphere locations computed from the Akhka MLS passes, ‘n’ column shows the number of visibility (passes) of each target in the MLS data.

**Target**		**Mean (m)**			**Standard Deviation (m)**			**Max. Absolute Error (m)**		
#	n	E	N	h	E	N	h	E	N	h
1	7	0.007	−0.011	−0.005	0.009	0.013	0.033	0.017	0.037	0.056
2	11	0.005	0.004	0.011	0.010	0.010	0.026	0.022	0.017	0.042
3	9	−0.005	−0.002	0.025	0.014	0.008	0.027	0.029	0.015	0.053
4	4	0.001	0.010	0.018	0.004	0.013	0.027	0.005	0.018	0.050
5	12	−0.004	0.011	0.028	0.013	0.016	0.040	0.024	0.030	0.089
6	15	−0.007	0.010	0.015	0.013	0.018	0.038	0.028	0.042	0.085
7	10	−0.009	0.005	−0.004	0.011	0.011	0.019	0.028	0.020	0.031
8	5	−0.005	0.017	0.054	0.013	0.015	0.020	0.018	0.035	0.077

Systematic errors										
		−0.003	0.006	0.018	0.009	0.009	0.019			
